# Supplementary material for: Developing the evidence and associated service models to support older adults living with frailty to manage their pain and to reduce its impact on their lives: protocol for a mixed-method, co-design study (The POPPY Study)
Source: BMJ Open. 2023 Jun 26;13(6):e074785. doi: 10.1136/bmjopen-2023-074785 (PMC10314646; doi:10.1136/bmjopen-2023-074785)
Supplement: Author's manuscript [file bmjopen-2023-074785.draft_revisions.pdf]

BMJ Open is committed to open peer review. As part of this commitment we make the peer review history of every article we publish publicly available.

When an article is published we post the peer reviewers' comments and the authors' responses online. We also post the versions of the paper that were used during peer review. These are the versions that the peer review comments apply to.

The versions of the paper that follow are the versions that were submitted during the peer review process. They are not the versions of record or the final published versions. They should not be cited or distributed as the published version of this manuscript.

BMJ Open is an open access journal and the full, final, typeset and author-corrected version of record of the manuscript is available on our site with no access controls, subscription charges or pay-per-view fees (http://bmjopen.bmj.com).

If you have any questions on BMJ Open's open peer review process please email
info.bmjopen@bmj.com

BMJ Open

Developing the evidence and associated service models to support older adults living with frailty to manage their pain and to reduce its impact on their lives: a mixed-method, co-design study (The POPPY Study)

Journal:	*BMJ Open*
Manuscript ID	bmjopen-2023-074785
Article Type:	Protocol
Date Submitted by the Author:	17-Apr-2023
Complete List of Authors:	Brown, Lesley; Bradford Institute for Health Research, Academic Unit for Ageing and Stroke Research<br>Mossabir , Rahena; Bradford Institute for Health Research, Academic Unit for Ageing and Stroke<br>Harrison , Nicola ; Bradford Institute for Health Research, Academic Unit for Ageing and Stroke Research<br>Lam, Natalie; University of York, , Department of Health Sciences<br>Williams, Amanda; University College London,<br>Antcliff, Deborah; Northern Care Alliance NHS Group, Physiotherapy Department; University of Leeds Faculty of Medicine and Health, School of Healthcare<br>Schofield, Patricia; University of Plymouth, School of Nursing & Midwifery<br>Clegg, Andrew; University of Leeds, Academic Unit of Elderly Care & Rehabilitation<br>Suleman, Asim.; Hollyns Health & Wellbeing<br>Grice, Anne; Bradford Institute for Health Research, Academic Unit for Ageing and Stroke Research<br>Forster, Anne; University of Leeds, Academic Unit of Elderly Care and Rehabilitat
Keywords:	PAIN MANAGEMENT, Aging, Chronic Pain

SCHOLARONE™
Manuscripts

Full title: Developing the evidence and associated service models to support older adults living with frailty to manage their pain and to reduce its impact on their lives: a mixed-method, co-design study (The POPPY Study).

Short title: Pain in Older adults with frailty - The POPPY Study.

ABSTRACT

Introduction

The POPPY study is a mixed-method, co-design study which aims to develop the content, implementation strategies, service and professional guidance to support older adults with frailty to manage their pain.

Methods and Analysis

Oversight is provided from a Patient and Public Involvement (PPI) group and an independent steering committee.

The study has four phases:

- Phase 1. Research evidence and information synthesis from randomised controlled trials of multicomponent pain management programmes and psychological therapies for community-dwelling older adults.
- Phase 2. Qualitative interviews with 30 community-dwelling older adults (≥75 years) living with frailty and persistent pain, including dyadic interviews with a spouse or unpaid carer.
- Phase 3. Qualitative interviews with healthcare professionals (HCPs) working within various pain service types; 5 – 8 HCPs per service and up to 12 services including primary care, secondary care, tertiary centres and services with voluntary sector input.
- Phase 4. Co-design workshops with older adults, HCPs and commissioners.

Inclusion criteria (Phase 2): community dwelling older adults (≥75 years) living with frailty and persistent pain.

Exclusion criteria (Phase 2): Care home residents, a dementia or cancer diagnosis. Cancer survivors, ≥ 5 years cancer free, and not undergoing active cancer treatment can participate.

Analysis: Phase 1 narrative synthesis. Phase 2 analysis will use grounded theory analysis. Phase 3 will use thematic analysis.

For peer review only - http://bmjopen.bmj.com/site/about/guidelines.xhtml

Ethics and Dissemination

The protocol was approved by Leeds-East Research Ethics Committee on 28/04/2022 (22/YH/0080). Consent is sought if an individual is willing to participate (Phases 2, 3 and 4) and has capacity.

The research will generate new knowledge and guidance for services to improve access and deliver targeted support to reduce the pain burden of older adults with frailty. Findings will be disseminated at conferences, in newsletters and journals and to Local Authorities and charities.

Strengths and Limitations

- A Patient and Public Involvement (PPI) group will contribute to all phases of the study. Members will be provided with appropriate training as required.
- The POPPY study will use a mixed method co-design approach, working with relevant stakeholders to develop service and professional guidance to support older adults with frailty to better manage their pain and reduce its negative impact on their lives.
- The study will recruit underserved communities including those with different levels of frailty. It will include people from different geographical locations, and from urban and rural settings, spanning the least deprived and most deprived communities.
- A range of different types of pain services will be invited to participate, including primary care, secondary care, specialist tertiary services and those with a voluntary sector component.
- Older adults with a diagnosis of dementia, a current cancer diagnosis, or living in a care home will not be eligible.

INTRODUCTION

Chronic pain - persistent pain of at least three months duration - is common amongst older adults as a consequence of arthritic and other diseases (1). Pain is more common in older adults with other chronic diseases (2). Prevalence ranges from 25% to 76%, depending on population sampled and methodology (1, 3). Pain contributes to disability and emotional distress (1, 3) and the cumulative and varied effects of pain worsen the pain itself and increase its impact on everyday life and mood (4). Pain is associated with disability from reduced mobility, restricted activity, falls, depression and anxiety, sleep impairment and isolation (1, 5). Pain is often under-recognised and undertreated in older adults (6). Addressing pain in older adults often requires a different approach compared to a younger person due to concomitant multimorbidity, polypharmacy and frailty (6). Barriers to pain management, relevant to older adults, draw on a limited evidence base to guide treatment

For peer review only

For peer review only - http://bmjopen.bmj.com/site/about/guidelines.xhtml

decisions, concerns about treatment-related harms, and older adults' beliefs about pain and how it should be managed (5).

Frailty is characterised by age-related decline across multiple physiological systems and vulnerability to disproportionate changes in health after relatively minor health events, such as a minor infection or a minor operation (7). The reported prevalence of frailty varies depending on the frailty models used (8), with prevalence of 12% and 24% reported among those of ≥50 years in a systematic review with data from 62 countries (1,755,497 participants) (8).

In the last 20 years, frailty has been conceptualised as an abnormal health state in relation to the ageing process. This has resulted in the development of robust models to identify and grade severity (9, 10). Frailty is considered a long-term condition requiring long-term strategies and interventions (11, 12). This has been facilitated by the ability of UK General Practices (GPs) to readily identify older adults living with frailty for appropriate services, using the electronic Frailty Index (eFI) now embedded within UK primary health care records (13)

Pain prevalence of between 31% and 60% was reported in a review of cohort and cross-sectional studies of community dwelling older adults with frailty (14). Older adults with frailty are about three times more likely to experience intrusive pain which interferes with normal work compared to fit older people (adjusted OR 3.53 (95% CI 2.47, 5.04)) (15) and pain impacts on mobility, ability to accomplish tasks, ability to socialise, and to sleep (15). Pain increases the risk of developing frailty in older adults with osteoarthritis (16) and a meta-analysis of pain and frailty from prospective longitudinal studies found that pain was associated with an increased risk of incident frailty and worsening frailty (17).

The presence of frailty identifies older adults with multimorbidity at high risk of falls, disability, hospitalisation and care home admission. Frailty negatively impacts on quality of life (18), caregiver burden and health and social care use (7). Older adults living with frailty are an '*underserved population*' with lower inclusion in research than expected from population estimates, and there is a particular neglect of how frail older adults respond to or engage with healthcare services compared to other groups (19). The healthcare burden of those with living with pain and frailty is not matched by the volume of research in this group. Pain, or pain impact, is potentially modifiable, and therefore makes an important target for services for older adults living with frailty.

METHODS AND ANALYSIS

AIM

For peer review only - http://bmjopen.bmj.com/site/about/guidelines.xhtml

To develop the content, delivery mode, implementation strategies, service and professional support and guidance to enable older adults with frailty to better manage their pain and reduce its negative impact on their lives, relationships, functioning and quality of life.

Objectives

1. Map research evidence and information from randomised controlled trials (RCTs) of multicomponent pain management programmes (PMPs) and psychological therapies, to develop hypotheses about processes and change mechanisms to inform the content and implementation strategies to meet the needs of older adults with frailty.

2. Undertake qualitative interviews with older adults living with pain and frailty to understand how they experience pain, engage with healthcare professionals (HCPs) about their pain and what service models are required to optimise access and provide support.

3. Undertake qualitative interviews with HCPs from a variety of pain services to identify opportunities and barriers to engaging older adults with pain and frailty into pain management services.

4. Develop and operationalise theory-informed pain management guidance specific to the needs of older adults with frailty in service contexts through co-design involving relevant stakeholders (HCPs, commissioners, older adults, relatives and carers).

Oversight Patient and Public Involvement Group (PPI)

PPI group members will meet quarterly to oversee the research programme and contribute to the development of resources and outputs. They will be offered basic training in research methods to enable them to make informed decisions and comments about the study.

The Oversight PPI Group will comprise up to eight members including those in later life living with pain, a spouse/family member or unpaid carer of an older person living with pain, and third sector representation. Members will join meetings in-person or remotely (video-conference). One group member (AG) is a grant co-applicant and will attend the Programme Management Group (PMG) meetings.

DESIGN

Mixed-method, co-design study with four phases:

- Phase 1 Review to map research evidence of PMPs and psychological therapies
- Phase 2 Qualitative interviews with older adults living with frailty and pain
- Phase 3 Qualitative interviews with HCPs working in a variety of pain service settings
- Phase 4 Co-design workshops (4 workshops in 3 locations).

See figure 1 for study flow diagram for the research programme.

For peer review only - http://bmjopen.bmj.com/site/about/guidelines.xhtml

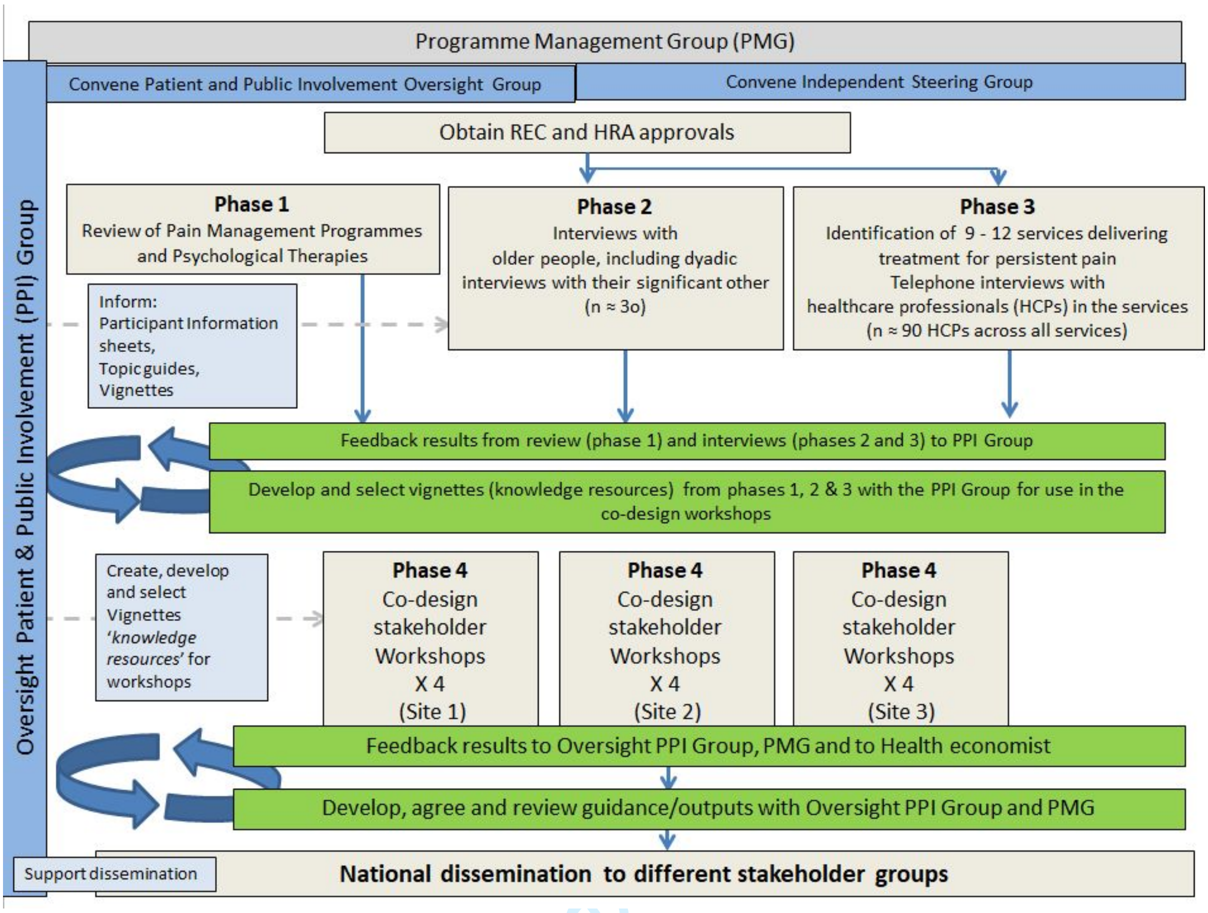

Figure1. Study flow diagram

PHASE 1: A REVIEW TO MAP RESEARCH EVIDENCE AND INFORMATION FROM RANDOMISED CONTROLLED TRIALS OF MULTICOMPONENT PAIN MANAGEMENT PROGRAMMES AND PSYCHOLOGICAL THERAPIES TARGETED COMMUNITY-DWELLING OLDER ADULTS.

In this literature review, we will identify multicomponent pain management programmes (PMPs) and Psychological Therapies (PTs) that have been conducted with older adults (study participants' mean age ≥ 65 years) in randomised controlled trials (published before June 2022). The information which we will collect and explore includes the intervention content (e.g., exercise, education), delivery mode (e.g., face-to-face, in group), pain dimensions addressed (e.g., physical, cognitive, affective), theoretical or conceptual framework, and which interventions are potentially effective for older adults with frailty to manage their pain. The methodology and results will be reported in detail elsewhere. The findings will inform subsequent phases of this project.

PHASE 2: QUALITATIVE INTERVIEWS WITH OLDER ADULTS LIVING WITH FRAILTY AND PAIN

Overview

For peer review only - http://bmjopen.bmj.com/site/about/guidelines.xhtml

Phase 2 will produce a detailed understanding of the experiences of older adults of living with pain and engaging with HCPs about their pain.

The negative effects of pain extend beyond the patient and can impact on relationships (5). Conversely, there is evidence of enhanced emotional wellbeing and reduced pain with spousal participation with some pain treatments (20). Therefore, some interviews will include the older person and her/his spouse/partner, family member (significant other) or unpaid carer. This will enable exploration of inter-dependent perspectives of individuals with pain and those close to them (21).

Interviews will be conducted on two occasions up to 10 weeks apart. Longitudinal studies using qualitative methods prioritise exploration of phenomena over time (22) and are helpful for exploring older adults thoughts and feelings. We do not anticipate capturing change in the pain experience in the 10-week timeframe. However, initial interviews can prompt reflection on the pain experience. Between interviews we will ask interviewees to note examples of how pain impinges on decision-making and action. This will be supported with materials to prompt note-taking. However, older adults can still participate in the study if they choose not to document their experiences of living with pain. The participants will be living with both frailty and pain and some may find it overly burdensome. If a participant chooses not to proceed, or is unable to proceed, with the second interview, we will seek permission to still retain and use the information already collected from the first interview.

Inclusion criteria

Participants will be recruited from The Community Ageing Research 75+ (CARE75+) study (ISRCTN16588124) (23, 24). CARE75+ is a longitudinal cohort study established in 2014 recruiting older adults (≥75 years). The study captures extensive observational data and provides a recruitment platform for research with older adults.

Participants will be ≥ 75 years and will include those living with frailty and with persistent pain. Frailty data will be captured from their latest available frailty assessment using either Fried frailty criteria (score 1, 2 = pre-frailty, 3, 4, 5 = frail) (10), or electronic frailty Index (eFI) score ≥0.13 (13).

Exclusion criteria

Care home residents, those with an estimated life expectancy of three months or less, or in receipt of palliative care, or those with a dementia diagnosis or a current cancer diagnosis will not be eligible. Cancer survivors, of ≥ 5 years cancer free and not undergoing active cancer treatment, are eligible.

Sample identification and recruitment for interviews with older adults

We will identify CARE75+ participants who have provided consent to be approached about other studies. Additionally, those undergoing routine CARE75+ assessments will be informed

For peer review only - http://bmjopen.bmj.com/site/about/guidelines.xhtml

about the study. CARE75+ participants will be invited that have previously indicated experiencing pain or pain impact during their routine CARE75+ assessment or are in receipt of medication prescribed for pain (e.g. co-codamol) or a condition associated with pain (e.g. osteoarthritis).

Eligibility screening to identify those currently meeting the criteria for persistent pain (25) will be conducted during the first telephone contact. See Figure 2. Researchers will make note of those invited that are not eligible, do not want to participate (and the reason if provided) and those unable to be contacted.

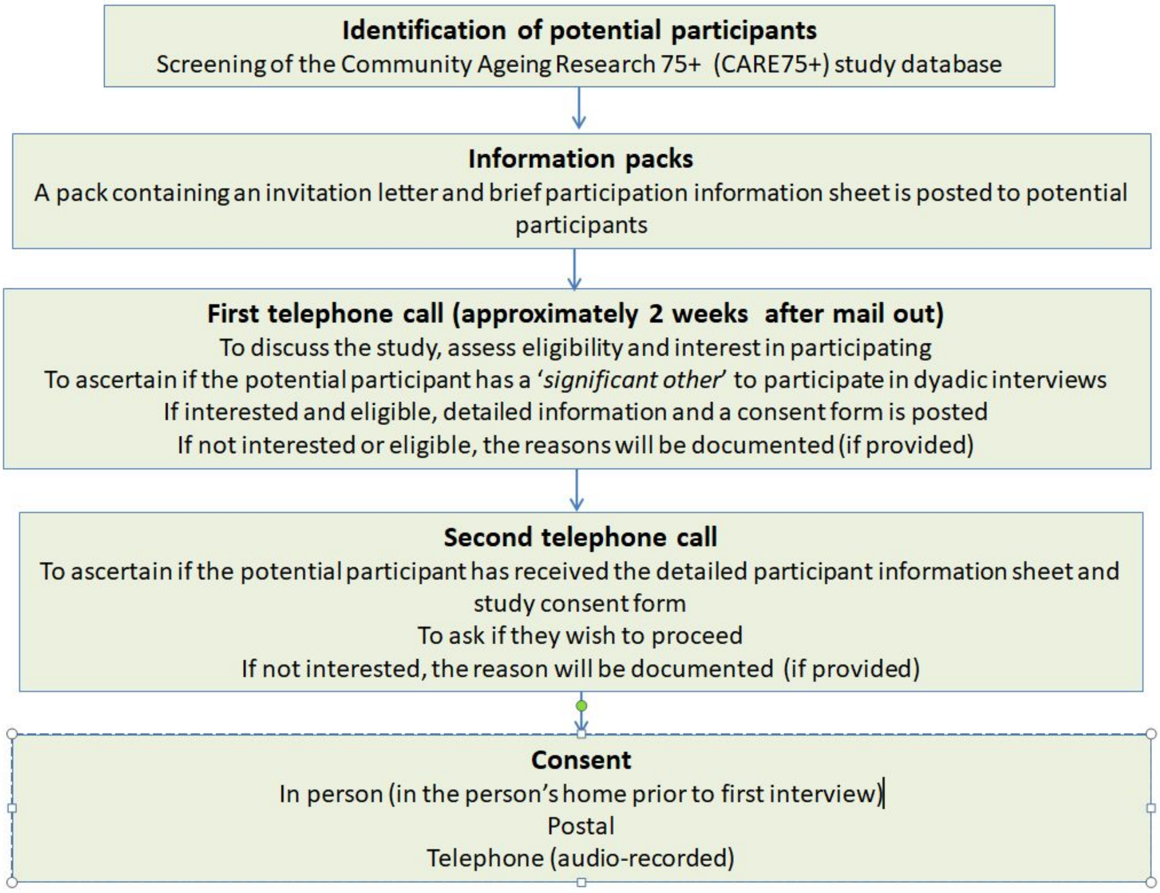

Figure 2. Identification of participants and recruitment flow diagram

Informed consent

Researchers will assess an individual's capacity to consent. Consent will be gained from those with capacity to make a decision about participating. Those who lack capacity will not

For peer review only - http://bmjopen.bmj.com/site/about/guidelines.xhtml

be included. Consent can be provided via a written consent form or verbally (audio recorded).

As well as mandatory consent items, the consent form will include optional items that will enable the researcher to: invite the participant's significant other or unpaid carer to participate in dyadic interviews (if applicable); invite the participant to the co-design workshops at a later date (phase 4); and to access participant information provided as part of the CARE75+ study assessments, for example, information on health conditions, mood, or quality of life. This information will help provide a contextual picture of the person, to supplement interview findings.

Separate consent will be sought from the significant other or unpaid carer to participate in dyadic interviews.

Sampling frame

We will interview up to 30 older adults. Some interviews will be dyadic, including a significant other or unpaid carer. We will purposively sample to include people from different geographical locations, from a mixture of urban and rural locations and spanning the least deprived and most deprived location. Deprivation levels will be ascertained from the English Index of Multiple Deprivation (IMD) data from post codes.

We will include people from different living circumstances (living alone/with spouse) and with different levels of frailty (10, 13). We will include people from different ethno-cultural backgrounds if feasible to do so mindful of language constraints. Furthermore, as we simultaneously collect and analyse data we will adopt theoretical sampling to support theory generation (26).

Topic guides

Topic guides will be informed from current pain management guidelines (e.g. National Institute for Health and Care Excellence) (27), PMG members and the PPI Oversight group. Topic guides will be piloted with PPI Oversight group members to ensure the questions prompt meaningful discussions. Topic guides will be revisited after initial interviews and adapted accordingly to reflect emerging topics.

Interviews

Interviews (10 weeks apart) will take place in the person's home, by telephone or videoconference depending on the participant's preference.

Data analysis

Interviews will be audio-recorded and transcribed verbatim. We will employ grounded theory analytic techniques (28) including simultaneous data collection and analysis, constant

For peer review only - http://bmjopen.bmj.com/site/about/guidelines.xhtml

comparison and search for negative cases and engage with research findings from the literature review mapping exercise (Phase 1). Coding will be managed using NVivo software V.12 (29).

Anonymous findings will be shared with the Oversight PPI group to identify case study examples that will be developed into vignettes and shared in Phase 3 and Phase 4 to inform discussions.

Sharing findings with dementia groups

We plan to share findings from Phase 2 with at least one community dementia group to get their comments and feedback.

Phase 3 Qualitative interviews with healthcare professionals (HCPs) working within a variety of pain services

We will identify up to 12 services that provide treatment for persistent pain (details of service identification below). The service manager (or designate) will complete a questionnaire about their service and they will identify HCPs to participate in qualitative interviews to understand the barriers and facilitators to engaging older adults with frailty into their service. This will provide an understanding of the perceptions, attitudes and barriers to engagement in pain services for older adults with comorbidities and with frailty. Data on what support healthcare professionals consider necessary to better support older adults living with pain and what resources might be most useful will be gathered.

Service identification

We will purposively select up to 12 pain services across three regions in England (Yorkshire, North West and the South West Peninsula). We will contact, Integrated Care Systems (ICS), Clinical Research Networks (CRNs), utilise Programme Management Group contacts, review data from previous pain service audits, and via online NHS directories to identify pain services in those areas in terms of organisational location (primary care/community care-based), and provider type (NHS, third sector, private). We will engage with commissioners and clinicians in the areas to understand if specific pain services have been commissioned or are under consideration for people with frailty. From the identified services we will purposively select a range of service types, organisational locations and providers (3 to 4 services within each of the three geographical locations) with a mixture of population characteristics.

Service questionnaire content

The service manager/service lead (or their designate) will complete a questionnaire to provide information about their service. The questionnaire will include questions on service setting and referral options, waiting times to access service, service aims, eligibility criteria, service exclusions, and modes of delivery, staff disciplines, and the characteristics of those

For peer review only - http://bmjopen.bmj.com/site/about/guidelines.xhtml

typically using the service (e.g. age, ethnicity, and inclusion of non-English speakers), the treatments provided and the accessibility of the service. There will be a mixture of open and close-ended questions.

Administration and analysis of the questionnaire

The service manager/service lead (or their designate) in each participating service will be asked to complete the questionnaire via an email link, using the survey tool, Redcap (30, 31). The questionnaire recipient will be asked to complete it within one month and prompted via an email if it is not completed within this time period. On submission of the completed questionnaire, data is automatically uploaded onto a secure server at Bradford Teaching Hospitals NHS Foundation Trust. Quantitative data from the demographic and closed-ended survey questions will be reported using descriptive statistics. Qualitative data from the open-ended questions will be analysed using thematic analysis (32).

Identification of HCPs for telephone interviews

We will conduct interviews with approximately 5-8 staff members in each participating service to gain an in-depth understanding of provision and professional perceptions of locating services for support with pain for older adults with frailty within local health systems. We will include psychologists, nurses, physiotherapists, occupational therapists, doctors and voluntary sector staff. We will aim for representation from the range of roles within each service, depending on the service type. The service manager/service lead (or their designate) will be asked to approach staff within the service for their initial expression of interest to participate in the interviews. Interviews will be conducted by telephone or teleconference. The cost of staff time will be reimbursed to the organisation.

Informed consent for interviews with HCPs within the service

The researcher will provide an information sheet and explain the purpose of the study. Staff will provide fully informed consent before they participate. Consent will be audio-recorded or the researcher can complete a consent form.

Topic guide for telephone interviews with HCPs

Interviews will cover perceptions, attitudes and barriers to engagement in pain services for older adults and particularly those with frailty. We will gather data on what support healthcare professionals consider necessary to better support older adults living with pain and what resources might be useful; the key components of pain services for older adults with comorbidities and with frailty; and what information commissioners need to know when developing/funding pain services for older adults with co-morbidities and frailty; what extra training for HCPs might be required; what other services need to be involved/linked in and how to improve accessibility.

For peer review only - http://bmjopen.bmj.com/site/about/guidelines.xhtml

Topic guides will be developed from on-going findings from phase 2 and with input from the Oversight PPI group and the PMG. Topic guides will be adapted if new topics emerge as the interviews progress.

We will include one or two case study vignettes to encourage discussion and reflection. The Oversight PPI group will help construct vignettes, developed from the older peoples' interviews or PPI members' experiences of living with pain, to understand how HCPs might manage a particular situation with an older person presenting with pain and frailty.

Analysis of HCP interviews

Interviews will be recorded and transcribed verbatim. We will employ an interpretive descriptive approach to analysis to construct a summary of professionals' accounts of their work in pain management. This approach is an Interpretative Phenomenological Analysis, a form of thematic analysis (32). Coding will be managed using NVivo software V.12(29).

Sharing findings with dementia groups

We plan to share findings from Phase 2 and Phase 3 with at least one community dementia group. Their comments and feedback will be shared with the stakeholder groups in the co-design workshops (Phase 4).

Phase 4 Co-design workshops with older adults, HCPs and commissioners

We will use a co-design approach (33) with multiple stakeholders to inform supportive interventions that are workable in different service contexts and that meet the specific needs of older adults living with frailty.

Workshop participants

We will establish stakeholder groups in the three different locations (Yorkshire, North West, South West Peninsular). Participants will include older adults, their significant other or unpaid caregiver, PPI member contacts, multidisciplinary professionals involved in pain services or services for older adults, service managers and commissioners. Some stakeholders will have participated in phases 2 and 3. All stakeholders will be provided with information and will provide informed consent, including those that have participated in earlier study phases.

Resources for workshops

Outputs from each of the empirical studies (Phases 1, 2, 3) will provide the vignettes or '*knowledge resources*' to facilitate discussion between stakeholder groups. This will iteratively inform the co-design work in sequential steps. The Oversight PPI Group will contribute towards the creation and selection of the knowledge resource vignettes for the

For peer review only - http://bmjopen.bmj.com/site/about/guidelines.xhtml

co-design workshops. This will include selecting key themes/topics from findings from Phases 2 and 3 they consider impactful and pertinent.

Workshop plan

We plan four workshops in the three participating sites. Workshops will be located to minimise travel burden. Workshops will last approximately two hours and include a refreshment break. Option to join the workshops remotely (by teleconference) will be available. Participants will receive participant information sheets prior to the workshops which will provide background study information and outline the workshop process.

Workshop 1

The first workshop will provide the background to the study, an 'ice-breaker' session and an opportunity to develop an understanding of the co-design process and to consider ways to work collaboratively.

Workshops 2 and 3

Participants will be asked to reflect on the knowledge resources generated from the previous study components. These will include narratives, illustrations or audio-visual podcasts.

Workshop 4

Participants will consider ideas about what changes in services are required, and what support is required to enable access to, and provide appropriate assistance with pain management to older adults living with frailty. This information will be combined with other sources of information, from early study phases to help identify barriers that will need to be overcome in each local setting to facilitate change, optimise service take up and provide better support.

COSTING OF A PAIN AND FRAILTY SERVICE

A health economist will undertake a costing analysis of a Pain and Frailty Service. This will consider costs of resource inputs associated with delivering the Pain and Frailty Service. Key inputs include staffing and training, service delivery and associated technology among other components of a good pain and frailty service as identified in objective 4. We anticipate a range of costing scenarios, depending on whether a completely new service or service components would be required, or if an established service could be extended.

OUTPUTS

Outputs from the three co-design groups will be summarised and presented to the Oversight PPI Group and the PMG. The agreed final outputs will draw on all layers of stakeholder work. Outputs will be a synthesis of sets of interventions, processes and

For peer review only - http://bmjopen.bmj.com/site/about/guidelines.xhtml

guidance for designing, commissioning and implementing pain management services for older adults with frailty. The primary outputs will include a Pain and Frailty Service Commissioning Pack.

Pain and Frailty Service Commissioning Pack

The Commissioning Pack will provide practical guidance for healthcare commissioners to facilitate and improve services for older adults living with frailty and pain. This will include:

- The case for change including the evidence and background to pain and frailty.
- Case study examples of living with frailty and pain.
- Examples of services successfully meeting the needs of older adults living with pain and frailty (if identified).
- Examples of HCPs working successfully with older adults with frailty and pain (if identified).
- How older adults should be identified and referred for services and different routes to optimise take-up.
- How services might be located within current services, or aligned to existing services.
- The components of a good frailty and pain service:
    - Format (group, individual, web-access)
    - Duration of participation in the service
    - Service components (e.g., peer support, education, exercise, coping strategies, psychological therapies)
    - Which healthcare professionals should be involved in delivering the service
    - How services might better support older adults from Black, Asian and minority ethnic communities (if differences emerge from the study findings)
    - How services might support people with different levels of frailty (if differences emerge from study findings).
- Case examples of local implementation guidance and how good practice might be adopted and customised for local needs.
- An outline of the potential costings of a new pain and frailty service or service components.

Discussion

The need to develop new models of care for older adults living with frailty is highlighted in the NHS Long Term Plan (12). In light of population ageing and the increasing numbers of people in advanced older age, targeted services addressing the specific needs of people with

For peer review only - http://bmjopen.bmj.com/site/about/guidelines.xhtml

frailty are required. Older adults living with frailty are more likely to live with pain and for pain to have more impact on their lives than for fitter older adults (15). Pain can contribute to worsening frailty and an increase in incident frailty (17). As such, pain makes a suitable target for services for older adults. To date, we do not know the best way to deliver services for older adults living with both frailty and pain, where services should be situated to optimise uptake and adherence and what the components of a good pain and frailty service should include.

Strengths and limitations

The POPPY study will use a mixed method co-design approach, working with relevant stakeholders to develop service and professional guidance to support older adults with frailty to better manage their pain A Patient and Public Involvement (PPI) group will contribute to all phases of the study. The POPPY study will include the views of an underserved group of older adults, i.e. those with frailty, often under-represented in research (19). Additionally, this study will include a range of different service models to understand the advantages and disadvantages of different service types to optimise take up and improve outcomes for this particular population.

Identifying POPPY participants from the CARE75+ cohort is an efficient and cost-effective way to identify a well-phenotyped population of older adults in terms of their frailty characteristics. The opportunity to capture additional information, from those that agree, from previously collected assessment data, including data on mobility, health conditions, loneliness and quality of life, will provide comprehensive contextual information to supplement interview findings. In-depth interviews will provide a detailed understanding of the impact of pain in this population, how they currently engage with services, and the reasons why some do not.

Some participants who participate in the interviews (phase 2) will be invited to co-design workshops (phase 4). Workshop participation will require a degree of engagement that may be beyond the scope of those living with dementia. As such those living with dementia have not been included in this study. However, we plan to share findings from Phase 2 and Phase 3 with at least one community dementia group to get their comments, insight and feedback. This will be shared with the stakeholder groups in the co-design workshops (Phase 4) so there is some recognition of the applicability of earlier findings to older adults living with dementia.

Older adults living in a care home or nursing home are not eligible for the POPPY study. We acknowledge that pain is an important issue, including for those living in care or nursing homes (34). However, the typical characteristics of care home residents, often including a high prevalence of dementia, dependence for basic activities of daily living and very limited

For peer review only - http://bmjopen.bmj.com/site/about/guidelines.xhtml

mobility, mean that service guidance would be different for this population. As such, this was beyond the scope of this study.

Furthermore, for Phase 2 of the POPPY study, older adults with a current cancer diagnosis are not eligible. Cancer survivors (at least 5 years cancer free and not undergoing active cancer treatment) can participate if they meet the eligibility criteria. Additionally, we will consult with the different services participating in Phase 3, on their guidelines for including cancer patients, or not, within their particular services.

The inclusion of a range of different pain services in different locations will enable us to consider what the best service models are to support older adults with frailty and pain. However, conducting research within NHS organisations presents challenges at the current time. This is partly as a legacy from COVID-19 and as a result of on-going capacity issues within NHS organisations. The POPPY study is a relatively low burden study for healthcare professionals to participate in, and the research team will work flexibly to accommodate service staff to minimise disruption to the service. Nevertheless, services are under pressure and some services may consider they lack the capacity to participate in research at this time due to service demands. As such, some pragmatic decisions may need to be made in terms of including those services that have the capacity to participate. We will make note of services that we approach that do not subsequently participate and the reasons why if these are provided.

ETHICS AND DISSEMINATION

The POPPY study is a mixed-method study with low risk to participants. The POPPY study protocol was approved by the 28 April 2022 from The Yorkshire & the Humber - Leeds East Research Ethics Committee (Reference: 22/YH/0080).

The research will generate new knowledge and associated guidance for interventions, implementation strategies and services to improve access and deliver targeted support to reduce the pain burden of older adults living with frailty. Findings will be disseminated in numerous formats such as conferences, newsletters and journal papers and to a variety of groups including Local Authorities, Age UK, the British Pain Society and charities including Versus Arthritis.

Contributors

LB: substantial contribution to the conception and design of the work; drafting the work and critical revisions; approval of final manuscript; accountable for all aspects of the work.
RM: substantial contribution to the conception and design of the work; drafting the work and critical revisions; approval of final manuscript; accountable for all aspects of the work.

For peer review only - http://bmjopen.bmj.com/site/about/guidelines.xhtml

NH: substantial contribution to the conception and design of the work; drafting the work and critical revisions; approval of final manuscript; accountable for all aspects of the work.
NL: substantial contribution to the conception and design of the work; drafting the work and critical revisions; approval of final manuscript; accountable for all aspects of the work.
AG: substantial contribution to the conception and design of the work; drafting the work and critical revisions; approval of final manuscript; accountable for all aspects of the work.
AC: substantial contribution to the conception and design of the work; drafting the work and critical revisions; approval of final manuscript; accountable for all aspects of the work.
AW: substantial contribution to the conception and design of the work; drafting the work and critical revisions; approval of final manuscript; accountable for all aspects of the work.
DA: substantial contribution to the conception and design of the work; drafting the work and critical revisions; approval of final manuscript; accountable for all aspects of the work.
PS: substantial contribution to the conception and design of the work; drafting the work and critical revisions; approval of final manuscript; accountable for all aspects of the work.
AS: substantial contribution to the conception and design of the work; drafting the work and critical revisions; approval of final manuscript; accountable for all aspects of the work.
AF: substantial contribution to the conception and design of the work; drafting the work and critical revisions; approval of final manuscript; accountable for all aspects of the work

Competing interests

Competing Interests

1. All support for the present manuscript (e.g., funding, provision of study materials, medical writing, article processing charges, etc.): The POPPY study is funded by the National Institute for Health & Care Research (NIHR). Payment made to institutions for percentage of time/time contribution: LB, RM, NH, NL, AG, AC, AW, DA, PS, AS, AF.

2. Grants or contracts from any entity (if not indicated in item #1 above).

DA is funded by a National Institute for Health and Care Research (NIHR) School for Primary Care Research post-doctoral award (project reference C075) and by a Chartered Society of Physiotherapy Charitable Trust post-doctoral award (Grant number PRF/21/POD01). Payments made to the Northern Care Alliance NHS Foundation Trust and University of Keele; AS has grant/contact with PEARN Charitable Trust. Payment made to University of Plymouth.

3. Royalties or licenses: none declared.

4. Consulting fees: none declared.

5. Payment or honoraria for lectures, presentations, speakers bureaus, manuscript writing or educational events. Royal College of General Practitioners Chair/Speaker fees to AS; Mibe Pharmaceuticals Speaker fees AS.

6. Payment for expert testimony: none declared.

For peer review only - http://bmjopen.bmj.com/site/about/guidelines.xhtml

7. Support for attending meetings and/or travel: none declared.

8. Patents planned, issued or pending: none declared.

9. Participation on a Data Safety Monitoring Board or Advisory Board: none declared.

10. Leadership or fiduciary role in other board, society, committee or advocacy group, paid or unpaid: MSK Champion Versus Arthritis Charity Leadership Role, AS.

11. Stock or stock options: none declared.

12. Receipt of equipment, materials, drugs, medical writing, gifts or other services: none declared.

13. Other financial or non-financial interests: none declared.

For peer review only

References

1. Abdulla A, Adams N, Bone M, et al. Guidance on the management of pain in older adults. *Age Ageing* 2013; 42:i1-57.

2. Van Hecke O, Torrance N, Smith B. Chronic pain epidemiology and its clinical relevance. *Br. J. Anaesth* 2013; 111:13-8.

3. Fayaz A, Croft P, Langford RM, et al. Prevalence of chronic pain in the UK: a systematic review and meta-analysis of population studies. *BMJ Open* 2016; 6(6):e010364.

4. Chou KL. Reciprocal relationship between pain and depression in older adults: evidence from the English Longitudinal Study of Ageing. J. Affect. Disord 2007; 102:115-23.

5. Reid MC, Eccleston C, Pillemer K. Management of chronic pain in older adults. *BMJ* 2015;350.

6. Ong T, Thiam CN. Special consideration for pain management in the older person. *Clinical Medicine* 2022; 22:295.

7. Clegg A, Young J, Iliffe S, et al. Frailty in elderly people. Lancet 2013; 381:752–62.

8. O'Caoimh R, Sezgin D, O'Donovan MR, et al. Prevalence of frailty in 62 countries across the world: a systematic review and meta-analysis of population-level studies *Age Ageing* 2021; 50:96-104.

9. Rockwood K, Song X, MacKnight C, et al. A global clinical measure of fitness and frailty in elderly people. *CMAJ* 2005; 173:489-95.

10. Fried LP, Tangen CM, Walston J, et al. Frailty in older adults: evidence for a phenotype. *J Gerontol A Biol Sci Med Sci* 2001;56:M146–57.

For peer review only - http://bmjopen.bmj.com/site/about/guidelines.xhtml

11. Harrison JK, Clegg A, Conroy SP, et al. Managing frailty as a long-term condition. *Age Ageing* 2015; 44:732–5.

12. Department of Health. NHS Long term plan 2019 London: Department of Health; 2019. Available: https://www.longtermplan.nhs.uk. [Accessed 2023 Feb 07].

13. Clegg A, Bates C, Young J et al. Development and validation of an electronic frailty index using routine primary care electronic health record data. Age and ageing. 2016; 45:353-60.

14. Brown L, Young J, Clegg A, et al. Pain in older adults with frailty. *Reviews in Clinical Gerontology*. 2015; 25:159-71.

15. Brown L, Young J, Teale E. A cross-sectional study of the impact of pain in older people with frailty: findings from the community ageing research 75+ (CARE75+) study. Adv Geriatr Med Res

2019; 1:e190002.

16. Veronese N, Maggi S, Trevisan C, et al. Pain Increases the Risk of Developing Frailty in Older Adults with Osteoarthritis. *Pain Med*. 2017 ;18:414-427. doi: 10.1093/pm/pnw163. PMID: 27497322.

17. Saraiva MD, Suzuki GS, Lin SM, et al. Persistent pain is a risk factor for frailty: a systematic review and meta-analysis from prospective longitudinal studies. *Age Ageing* 2018; 47:785-93.

18. Crocker TF, Brown L, Clegg A, et al. Quality of life is substantially worse for community-dwelling older adults living with frailty: Systematic review and meta-analysis. *Quality of Life Research*. 2019:1-16.

19. National Institute for Health Research. Improving inclusion of under-served groups in clinical research: Guidance from INCLUDE project, 2020. Available: https://www.nihr.ac.uk/documents/improving-inclusion-of-under-served-groups-in-clinical-research-guidance-from-include-project/25435. [Accessed 2023 Feb 07].

20. Keefe FJ, Blumenthal J, Baucom D, et al. Effects of spouse-assisted coping skills training and exercise training in patients with osteoarthritic knee pain: a randomized controlled study. *Pain* 2004; 110:539-49.

21. Hackett J, Godfrey M, Bennett MI. Patient and caregiver perspectives on managing pain in advanced cancer: a qualitative longitudinal study. Palliative Medicine 2016; 30:711-9.

For peer review only - http://bmjopen.bmj.com/site/about/guidelines.xhtml

22. Calman L, Brunton L, Molassiotis A. Developing longitudinal qualitative designs: lessons learned and recommendations for health services research. BMC medical research methodology. 2013; 13:1-10.

23. Heaven A, Brown L, Young J, et al. Community ageing research 75+ study (CARE75+): an experimental ageing and frailty research cohort. *BMJ Open* 2019; 9:e026744.

24. Brown L, Heaven A, Quinn C, et al. Community Ageing Research 75+ (CARE75+) REMOTE study: a remote model of recruitment and assessment of the health, well-being and social circumstances of older adults. *BMJ Open*. 2021;11:e048524.

25. Treede R-D, Rief W, Barke A, et al. A classification of chronic pain for ICD-11. *Pain*. 2015; 156:1003.

26. Glaser BG, Strauss AL. The discovery of grounded theory: Strategies for qualitative research. Routledge; 2017.

27. National Institute for Health and Care Excellence (NICE). Chronic pain (primary and secondary) in over 16s: assessment of all chronic pain and management of chronic primary pain. London: 2021 [Accessed 2023 Feb 07]. Available from: https://www.nice.org.uk/guidance/ng193

28. Corbin, J Strauss, A. Basics of qualitative research. 3rd ed. London: Sage; 2008.

29. QSR International Pty Ltd. (2018) NVivo (Version 12), https://www.qsrinternational.com/nvivo-qualitative-data-analysis-software/home.

30. Harris PA, Taylor R, Thielke R, et al. Research electronic data capture (REDCap)—a metadata-driven methodology and workflow process for providing translational research informatics support. *Journal of biomedical informatics*. 2009; 42(2):377-81.

31. Harris PA, Taylor R, Minor BL, et al. The REDCap consortium: Building an international community of software platform partners. *Journal of biomedical informatics*. 2019; 95:103208.

32. Braun V, Clarke V. Using thematic analysis in psychology. *Qualitative research in psychology*. 2006; 3:77-101.

33. Ziebland S, Coulter A, Calabrese JD, Locock L. Editors. Understanding and using health experiences: improving patient experience. Oxford: Oxford University Press; c2013. Chapter 9, Participatory action research: using experience based co-design (EBCD) to improve healthcare services.

34. Schofield P. The assessment of pain in older people: UK national guidelines. *Age Ageing*. 2018 Mar 1; 47(suppl_1):i1-22.

For peer review only - http://bmjopen.bmj.com/site/about/guidelines.xhtml

For peer review only

For peer review only - http://bmjopen.bmj.com/site/about/guidelines.xhtml

BMJ Open

Developing the evidence and associated service models to support older adults living with frailty to manage their pain and to reduce its impact on their lives: protocol for a mixed-method, co-design study (The POPPY Study)

Journal:	*BMJ Open*
Manuscript ID	bmjopen-2023-074785.R1
Article Type:	Protocol
Date Submitted by the Author:	04-Jun-2023
Complete List of Authors:	Brown, Lesley; Bradford Institute for Health Research, Academic Unit for Ageing and Stroke Research<br>Mossabir , Rahena; Bradford Institute for Health Research, Academic Unit for Ageing and Stroke<br>Harrison , Nicola ; Bradford Institute for Health Research, Academic Unit for Ageing and Stroke Research<br>Lam, Natalie; University of York, , Department of Health Sciences<br>Grice, Anne; Bradford Institute for Health Research, Academic Unit for Ageing and Stroke Research<br>Clegg, Andrew; University of Leeds, Academic Unit of Elderly Care & Rehabilitation<br>Williams, Amanda; University College London,<br>Antcliff, Deborah; Bury Integrated Pain Service, Bury Care Organisation, Northern Care Alliance NHS Foundation Trust; Keele University, School of Medicine<br>Schofield, Patricia; University of Plymouth, School of Nursing & Midwifery<br>Suleman, Asim.; Hollyns Health & Wellbeing<br>Forster, Anne; University of Leeds, Academic Unit of Elderly Care and Rehabilitat
**Primary Subject Heading**:	Health services research
Secondary Subject Heading:	Health services research
Keywords:	PAIN MANAGEMENT, Aging, Chronic Pain

SCHOLARONE™
Manuscripts

Developing the evidence and associated service models to support older adults living with frailty to manage their pain and to reduce its impact on their lives: protocol for a mixed-method, co-design study (The POPPY Study)

Authors

1. Lesley Brown, Academic Unit for Ageing and Stroke Research, Bradford Institute for Health Research, Bradford, UK.

2. Rahena Mossabir, Academic Unit for Ageing and Stroke Research, Bradford Institute for Health Research, Bradford, UK.

3. Nicola Harrison, Academic Unit for Ageing and Stroke Research, Bradford Institute for Health Research, Bradford, UK.

4. Natalie Lam, Department of Health Sciences, Faculty of Science, University of York, York, UK.

5. Anne Grice, The POPPY Study Oversight Patient and Public Involvement Group, Bradford Institute for Health Research, Bradford, UK.

6. Andrew Clegg, Academic Unit for Ageing and Stroke Research, University of Leeds, Faculty of Medicine and Health, Leeds, UK.

7. Amanda Williams, Research Department of Clinical, Educational & Health Psychology, University College London, London, UK.

8. Deborah Antcliff , Institution 1: Bury Integrated Pain Service, Bury Care Organisation, Northern Care Alliance NHS Foundation Trust, Bury, UK; Institution 2: School of Medicine, Keele University, Keele, UK; Institution 3: School of Healthcare, University of Leeds, Leeds, UK.

9. Pat Schofield, School of Nursing & Midwifery. Faculty of Health: Medicine, Dentistry and Human Sciences, University of Plymouth, Plymouth, UK.

10 Asim Suleman, Hollyns Health & Wellbeing, Bradford, UK.

11. Anne Forster, Academic Unit for Ageing and Stroke Research, University of Leeds, Faculty of Medicine and Health, Leeds, UK.

Corresponding author: Lesley Brown, Academic Unit for Ageing and Stroke Research, Temple Bank House, Bradford Royal Infirmary, Duckworth Lane, Bradford BD9 6RJ, UK. Lesley.brown@bthft.nhs.uk.

Key words: Pain, Ageing, Frailty

Word count: 5666

For peer review only - http://bmjopen.bmj.com/site/about/guidelines.xhtml

ABSTRACT

Introduction

The POPPY study is a mixed-method, co-design study which aims to develop the content, implementation strategies, service and professional guidance to support older adults with frailty to manage their pain.

Methods and analysis

The study has four phases: Phase 1, Research evidence and information synthesis from randomised controlled trials of multicomponent pain management programmes and psychological therapies for community-dwelling older adults. Phase 2, Qualitative interviews with 30 community-dwelling older adults (≥75 years) living with frailty and persistent pain, including dyadic interviews with a spouse or unpaid carer. Phase 3, Qualitative interviews with healthcare professionals (HCPs) working within various pain service types; 5 – 8 HCPs per service and up to 12 services including primary care, secondary care, tertiary centres and services with voluntary sector input. Phase 4, Co-design workshops with older adults, HCPs and commissioners. Inclusion criteria (Phase 2): community dwelling older adults (≥75 years) living with frailty and persistent pain. Exclusion criteria (Phase 2): Care home residents, a dementia or cancer diagnosis. Cancer survivors, ≥ 5 years cancer free, and not undergoing active cancer treatment can participate. Analysis for Phase 1 will use narrative synthesis, Phase 2 will use grounded theory analysis and Phase 3 will use thematic analysis. Oversight is provided from a Patient and Public Involvement (PPI) group and an independent steering committee.

Ethics and dissemination

The protocol was approved by Leeds-East Research Ethics Committee on 28/04/2022 (22/YH/0080). Consent is sought if an individual is willing to participate (Phases 2, 3 and 4) and has capacity. Findings will be disseminated at conferences, in newsletters and journals and to Local Authorities and charities.

For peer review only

Strengths and limitations of this study

- A Patient and Public Involvement (PPI) group will contribute to all phases of the study.
- The POPPY study will use a mixed method, co-design approach, working with relevant stakeholders to develop service and professional guidance to support older

For peer review only - http://bmjopen.bmj.com/site/about/guidelines.xhtml

adults with frailty to better manage their pain and reduce its negative impact on their lives.

- The study will include older adults with different levels of frailty and from the least deprived and most deprived communities.
- A range of different types of pain services will be invited to participate, including primary care, secondary care, specialist tertiary services and those with a voluntary sector component.
- Older adults with a diagnosis of dementia, a current cancer diagnosis, or living in a care home will not be eligible.

INTRODUCTION

Chronic pain - persistent pain of at least three months duration - is common amongst older adults as a consequence of arthritic and other diseases (1). Pain is more common in older adults with other chronic diseases (2). Prevalence ranges from 25% to 76%, depending on population sampled and methodology (1, 3). Pain contributes to disability and emotional distress (1, 3) and the cumulative and varied effects of pain worsen the pain itself and increase its impact on everyday life and mood (4). Pain is associated with disability from reduced mobility, restricted activity, falls, depression and anxiety, sleep impairment and isolation (1, 5). Pain is often under-recognised and undertreated in older adults (6). Addressing pain in older adults often requires a different approach compared to a younger person due to concomitant multimorbidity, polypharmacy and frailty (6). Barriers to pain management, relevant to older adults, draw on a limited evidence base to guide treatment decisions, concerns about treatment-related harms, and older adults' beliefs about pain and how it should be managed (5).

Frailty is characterised by age-related decline across multiple physiological systems and vulnerability to disproportionate changes in health after relatively minor health events, such as a minor infection or a minor operation (7). The reported prevalence of frailty varies depending on the frailty models used (8), with prevalence of 12% and 24% reported among those of ≥50 years in a systematic review with data from 62 countries (1,755,497 participants) (8).

In the last 20 years, frailty has been conceptualised as an abnormal health state in relation to the ageing process. This has resulted in the development of robust models to identify and grade severity (9, 10). Frailty is considered a long-term condition requiring long-term strategies and interventions (11, 12). This has been facilitated by the ability of UK General Practices (GPs) to readily identify older adults living with frailty for appropriate services,

For peer review only - http://bmjopen.bmj.com/site/about/guidelines.xhtml

using the electronic Frailty Index (eFI) now embedded within UK primary health care records (13)

Pain prevalence of between 31% and 60% was reported in a review of cohort and cross-sectional studies of community dwelling older adults with frailty (14). Older adults with frailty are about three times more likely to experience intrusive pain which interferes with normal work compared to fit older people (adjusted OR 3.53 (95% CI 2.47, 5.04)) (15) and pain impacts on mobility, ability to accomplish tasks, ability to socialise, and to sleep (15). Pain increases the risk of developing frailty in older adults with osteoarthritis (16) and a meta-analysis of pain and frailty from prospective longitudinal studies found that pain was associated with an increased risk of incident frailty and worsening frailty (17).

The presence of frailty identifies older adults with multimorbidity at high risk of falls, disability, hospitalisation and care home admission. Frailty negatively impacts on quality of life (18), caregiver burden and health and social care use (7). Older adults living with frailty are an '*underserved population*' with lower inclusion in research than expected from population estimates, and there is a particular neglect of how frail older adults respond to or engage with healthcare services compared to other groups (19). The healthcare burden of those with living with pain and frailty is not matched by the volume of research in this group. Pain, or pain impact, is potentially modifiable, and therefore makes an important target for services for older adults living with frailty.

METHODS AND ANALYSIS

Aim

To develop the content, delivery mode, implementation strategies, service and professional support and guidance to enable older adults with frailty to better manage their pain and reduce its negative impact on their lives, relationships, functioning and quality of life.

Objectives

1. Map research evidence and information from randomised controlled trials (RCTs) of multicomponent pain management programmes (PMPs) and psychological therapies, to develop hypotheses about processes and change mechanisms to inform the content and implementation strategies to meet the needs of older adults with frailty.

2. Undertake qualitative interviews with older adults living with pain and frailty to understand how they experience pain, engage with healthcare professionals (HCPs) about their pain and what service models are required to optimise access and provide support.

For peer review only - http://bmjopen.bmj.com/site/about/guidelines.xhtml

3. Undertake qualitative interviews with HCPs from a variety of pain services to identify opportunities and barriers to engaging older adults with pain and frailty into pain management services.

4. Develop and operationalise theory-informed pain management guidance specific to the needs of older adults with frailty in service contexts through co-design involving relevant stakeholders (HCPs, commissioners, older adults, relatives and carers).

Patient and public involvement

Patient and Public Involvement (PPI) group members will meet quarterly to oversee the research programme and contribute to the development of resources and outputs. They will be offered basic training in research methods to enable them to make informed decisions and comments about the study.

The Oversight PPI Group will comprise up to eight members including those in later life living with pain, a spouse/family member or unpaid carer of an older person living with pain, and third sector representation. Members will join meetings in-person or remotely (video-conference). One group member (AG) is a grant co-applicant and will attend the Programme Management Group (PMG) meetings.

Design

Mixed-method, co-design study with four phases:

- Phase 1 Review to map research evidence of PMPs and psychological therapies
- Phase 2 Qualitative interviews with older adults living with frailty and pain
- Phase 3 Qualitative interviews with HCPs working in a variety of pain service settings
- Phase 4 Co-design workshops (4 workshops in 3 locations).

See Figure 1.

The study started in April 2022 and will run until March 2025. Phase 1 was completed in May 2023. Recruitment for Phase 2 started in August 2022 and is on-going. Recruitment for Phase 3 started in October 2022 and is on-going.

For peer review only - http://bmjopen.bmj.com/site/about/guidelines.xhtml

Phase 1: A review to map research evidence and information from randomised controlled trials of multicomponent pain management programmes and psychological therapies targeted community-dwelling older adults

In this literature review, we will identify multicomponent pain management programmes (PMPs) and Psychological Therapies (PTs) that have been conducted with older adults (study participants' mean age ≥ 65 years) in randomised controlled trials (published before June 2022). The information which we will collect and explore includes the intervention content (e.g., exercise, education), delivery mode (e.g., face-to-face, in group), pain dimensions addressed (e.g., physical, cognitive, affective), theoretical or conceptual framework, and which interventions are potentially effective for older adults with frailty to manage their pain. The methodology and results will be reported in detail elsewhere. The findings will inform subsequent phases of this project.

Phase 2: Qualitative interviews with older adults living with frailty and pain

Overview

Phase 2 will produce a detailed understanding of the experiences of older adults of living with pain and engaging with HCPs about their pain.

The negative effects of pain extend beyond the patient and can impact on relationships (5). Conversely, there is evidence of enhanced emotional wellbeing and reduced pain with spousal participation with some pain treatments (20). Therefore, some interviews will include the older person and her/his spouse/partner, family member (significant other) or unpaid carer. This will enable exploration of inter-dependent perspectives of individuals with pain and those close to them (21).

Interviews will be conducted on two occasions up to 10 weeks apart. Longitudinal studies using qualitative methods prioritise exploration of phenomena over time (22) and are helpful for exploring older adults thoughts and feelings. We do not anticipate capturing change in the pain experience in the 10-week timeframe. However, initial interviews can prompt reflection on the pain experience. Between interviews we will ask interviewees to note examples of how pain impinges on decision-making and action. This will be supported with materials to prompt note-taking. However, older adults can still participate in the study if they choose not to document their experiences of living with pain. The participants will be living with both frailty and pain and some may find it overly burdensome. If a participant chooses not to proceed, or is unable to proceed, with the second interview, we will seek permission to still retain and use the information already collected from the first interview.

Inclusion criteria

Participants will be recruited from The Community Ageing Research 75+ (CARE75+) study (ISRCTN16588124) (23, 24). CARE75+ is a longitudinal cohort study established in 2014

For peer review only - http://bmjopen.bmj.com/site/about/guidelines.xhtml

recruiting older adults (≥75 years). The study captures extensive observational data and provides a recruitment platform for research with older adults.

Participants will be ≥ 75 years and will include those living with frailty and with persistent pain. Frailty data will be captured from their latest available frailty assessment using either Fried frailty criteria (score 1, 2 = pre-frailty, 3, 4, 5 = frail) (10), or electronic frailty Index (eFI) score ≥0.13 (13).

Exclusion criteria

Care home residents, those with an estimated life expectancy of three months or less, or in receipt of palliative care, or those with a dementia diagnosis or a current cancer diagnosis will not be eligible. Cancer survivors, of ≥ 5 years cancer free and not undergoing active cancer treatment, are eligible.

Sample identification and recruitment for interviews with older adults

We will identify CARE75+ participants who have provided consent to be approached about other studies. Additionally, those undergoing routine CARE75+ assessments will be informed about the study. CARE75+ participants will be invited that have previously indicated experiencing pain or pain impact during their routine CARE75+ assessment or are in receipt of medication prescribed for pain (e.g. co-codamol) or a condition associated with pain (e.g. osteoarthritis).

Eligibility screening to identify those currently meeting the criteria for persistent pain (25) will be conducted during the first telephone contact. See Figure 2. Researchers will make note of those invited that are not eligible, do not want to participate (and the reason if provided) and those unable to be contacted.

Informed consent

Researchers will assess an individual's capacity to consent. Consent will be gained from those with capacity to make a decision about participating. Those who lack capacity will not be included. Consent can be provided via a written consent form or verbally (audio recorded).

As well as mandatory consent items, the consent form will include optional items that will enable the researcher to: invite the participant's significant other or unpaid carer to participate in dyadic interviews (if applicable); invite the participant to the co-design workshops at a later date (phase 4); and to access participant information provided as part of the CARE75+ study assessments, for example, information on health conditions, mood, or quality of life. This information will help provide a contextual picture of the person, to supplement interview findings.

For peer review only - http://bmjopen.bmj.com/site/about/guidelines.xhtml

Separate consent will be sought from the significant other or unpaid carer to participate in dyadic interviews.

Sampling frame

We will interview up to 30 older adults. Some interviews will be dyadic, including a significant other or unpaid carer. We will purposively sample to include people from different geographical locations, from a mixture of urban and rural locations and spanning the least deprived and most deprived location. Deprivation levels will be ascertained from the English Index of Multiple Deprivation (IMD) data from post codes.

We will include people from different living circumstances (living alone/with spouse) and with different levels of frailty (10, 13). We will include people from different ethno-cultural backgrounds if feasible to do so mindful of language constraints. Furthermore, as we simultaneously collect and analyse data we will adopt theoretical sampling to support theory generation (26).

Topic guides

Topic guides will be informed from current pain management guidelines (e.g. National Institute for Health and Care Excellence) (27), PMG members and the PPI Oversight group. Topic guides will be piloted with PPI Oversight group members to ensure the questions prompt meaningful discussions. Topic guides will be revisited after initial interviews and adapted accordingly to reflect emerging topics.

Interviews

Interviews (up to 10 weeks apart) will take place in the person's home, by telephone or videoconference depending on the participant's preference.

Data analysis

Interviews will be audio-recorded and transcribed verbatim. We will employ grounded theory analytic techniques (28) including simultaneous data collection and analysis, constant comparison and search for negative cases and engage with research findings from the literature review mapping exercise (Phase 1). Coding will be managed using NVivo software V.12 (29).

Anonymous findings will be shared with the Oversight PPI group to identify case study examples that will be developed into vignettes and shared in Phase 3 and Phase 4 to inform discussions.

Sharing findings with dementia groups

We plan to share findings from Phase 2 with at least one community dementia group to get their comments and feedback.

For peer review only - http://bmjopen.bmj.com/site/about/guidelines.xhtml

Phase 3: Qualitative interviews with healthcare professionals (HCPs) working within a variety of pain services

We will identify up to 12 services that provide treatment for persistent pain (details of service identification below). The service manager (or designate) will complete a questionnaire about their service and they will identify HCPs to participate in qualitative interviews to understand the barriers and facilitators to engaging older adults with frailty into their service. This will provide an understanding of the perceptions, attitudes and barriers to engagement in pain services for older adults with comorbidities and with frailty. Data on what support healthcare professionals consider necessary to better support older adults living with pain and what resources might be most useful will be gathered.

Service identification

We will purposively select up to 12 pain services across three regions in England (Yorkshire, North West and the South West Peninsula). We will contact, Integrated Care Systems (ICS), Clinical Research Networks (CRNs), utilise Programme Management Group contacts, review data from previous pain service audits, and via online NHS directories to identify pain services in those areas in terms of organisational location (primary care/community care-based), and provider type (NHS, third sector, private). We will engage with commissioners and clinicians in the areas to understand if specific pain services have been commissioned or are under consideration for people with frailty. From the identified services we will purposively select a range of service types, organisational locations and providers (3 to 4 services within each of the three geographical locations) with a mixture of population characteristics.

Service questionnaire content

The service manager/service lead (or their designate) will complete a questionnaire to provide information about their service. The questionnaire will include questions on service setting and referral options, waiting times to access service, service aims, eligibility criteria, service exclusions, and modes of delivery, staff disciplines, and the characteristics of those typically using the service (e.g. age, ethnicity, and inclusion of non-English speakers), the treatments provided and the accessibility of the service. There will be a mixture of open and close-ended questions.

Administration and analysis of the questionnaire

The service manager/service lead (or their designate) in each participating service will be asked to complete the questionnaire via an email link, using the survey tool, Redcap (30, 31). The questionnaire recipient will be asked to complete it within one month and prompted via an email if it is not completed within this time period. On submission of the completed questionnaire, data is automatically uploaded onto a secure server at Bradford Teaching Hospitals NHS Foundation Trust. Quantitative data from the demographic and

For peer review only - http://bmjopen.bmj.com/site/about/guidelines.xhtml

closed-ended survey questions will be reported using descriptive statistics. Qualitative data from the open-ended questions will be analysed using thematic analysis (32).

Identification of HCPs for telephone interviews

We will conduct interviews with approximately 5-8 staff members in each participating service to gain an in-depth understanding of provision and professional perceptions of locating services for support with pain for older adults with frailty within local health systems. We will include psychologists, nurses, physiotherapists, occupational therapists, doctors and voluntary sector staff. We will aim for representation from the range of roles within each service, depending on the service type. The service manager/service lead (or their designate) will be asked to approach staff within the service for their initial expression of interest to participate in the interviews. Interviews will be conducted by telephone or teleconference. The cost of staff time will be reimbursed to the organisation.

Informed consent for interviews with HCPs within the service

The researcher will provide an information sheet and explain the purpose of the study. Staff will provide fully informed consent before they participate. Consent will be audio-recorded or the participant can complete a consent form.

Topic guide for telephone interviews with HCPs

Interviews will cover perceptions, attitudes and barriers to engagement in pain services for older adults and particularly those with frailty. We will gather data on what support healthcare professionals consider necessary to better support older adults living with pain and what resources might be useful; the key components of pain services for older adults with comorbidities and with frailty; and what information commissioners need to know when developing/funding pain services for older adults with co-morbidities and frailty; what extra training for HCPs might be required; what other services need to be involved/linked in and how to improve accessibility.

Topic guides will be developed from on-going findings from phase 2 and with input from the Oversight PPI group and the PMG. Topic guides will be adapted if new topics emerge as the interviews progress.

We will include one or two case study vignettes to encourage discussion and reflection. The Oversight PPI group will help construct vignettes, developed from the older peoples' interviews or PPI members' experiences of living with pain, to understand how HCPs might manage a particular situation with an older person presenting with pain and frailty.

Analysis of HCP interviews

Interviews will be recorded and transcribed verbatim. We will employ an interpretive descriptive approach to analysis to construct a summary of professionals' accounts of their

For peer review only - http://bmjopen.bmj.com/site/about/guidelines.xhtml

work in pain management. This approach is an Interpretative Phenomenological Analysis, a form of thematic analysis (32). Coding will be managed using NVivo software V.12(29).

Sharing findings with dementia groups

We plan to share findings from Phase 2 and Phase 3 with at least one community dementia group. Their comments and feedback will be shared with the stakeholder groups in the co-design workshops (Phase 4).

PHASE 4: CO-DESIGN WORKSHOPS WITH OLDER ADULTS, HCPS AND COMMISSIONERS

We will use a co-design approach (33) with multiple stakeholders to inform supportive interventions that are workable in different service contexts and that meet the specific needs of older adults living with frailty.

Workshop participants

We will establish stakeholder groups in the three different locations (Yorkshire, North West, South West Peninsular). Participants will include older adults, their significant other or unpaid caregiver, PPI member contacts, multidisciplinary professionals involved in pain services or services for older adults, service managers and commissioners. Some stakeholders will have participated in phases 2 and 3. All stakeholders will be provided with information and will provide informed consent, including those that have participated in earlier study phases.

Resources for workshops

Outputs from each of the empirical studies (Phases 1, 2, 3) will provide the vignettes or '*knowledge resources*' to facilitate discussion between stakeholder groups. This will iteratively inform the co-design work in sequential steps. The Oversight PPI Group will contribute towards the creation and selection of the knowledge resource vignettes for the co-design workshops. This will include selecting key themes/topics from findings from Phases 2 and 3 they consider impactful and pertinent.

Workshop plan

We plan four workshops in the three participating sites. Workshops will be located to minimise travel burden. Workshops will last approximately two hours and include a refreshment break. Option to join the workshops remotely (by teleconference) will be available. Participants will receive participant information sheets prior to the workshops which will provide background study information and outline the workshop process.

Workshop 1

For peer review only - http://bmjopen.bmj.com/site/about/guidelines.xhtml

The first workshop will provide the background to the study, an 'ice-breaker' session and an opportunity to develop an understanding of the co-design process and to consider ways to work collaboratively.

Workshops 2 and 3

Participants will be asked to reflect on the knowledge resources generated from the previous study components. These will include narratives, illustrations or audio-visual podcasts.

Workshop 4

Participants will consider ideas about what changes in services are required, and what support is required to enable access to, and provide appropriate assistance with pain management to older adults living with frailty. This information will be combined with other sources of information, from early study phases to help identify barriers that will need to be overcome in each local setting to facilitate change, optimise service take up and provide better support.

Costing of a pain and frailty service

A health economist will undertake a costing analysis of a Pain and Frailty Service. This will consider costs of resource inputs associated with delivering the Pain and Frailty Service. Key inputs include staffing and training, service delivery and associated technology among other components of a good pain and frailty service as identified in objective 4. We anticipate a range of costing scenarios, depending on whether a completely new service or service components would be required, or if an established service could be extended.

Outputs

Outputs from the three co-design groups will be summarised and presented to the Oversight PPI Group and the PMG. The agreed final outputs will draw on all layers of stakeholder work. Outputs will be a synthesis of sets of interventions, processes and guidance for designing, commissioning and implementing pain management services for older adults with frailty. The primary outputs will include a Pain and Frailty Service Commissioning Pack.

Pain and frailty service Commissioning pack

The Commissioning Pack will provide practical guidance for healthcare commissioners to facilitate and improve services for older adults living with frailty and pain. This will include:

- The case for change including the evidence and background to pain and frailty.
- Case study examples of living with frailty and pain.

For peer review only - http://bmjopen.bmj.com/site/about/guidelines.xhtml

- Examples of services successfully meeting the needs of older adults living with pain and frailty (if identified).
- Examples of HCPs working successfully with older adults with frailty and pain (if identified).
- How older adults should be identified and referred for services and different routes to optimise take-up.
- How services might be located within current services, or aligned to existing services.
- The components of a good frailty and pain service:
    - Format (group, individual, web-access)
    - Duration of participation in the service
    - Service components (e.g., peer support, education, exercise, coping strategies, psychological therapies)
    - Which healthcare professionals should be involved in delivering the service
    - How services might better support older adults from Black, Asian and minority ethnic communities (if differences emerge from the study findings)
    - How services might support people with different levels of frailty (if differences emerge from study findings).
- Case examples of local implementation guidance and how good practice might be adopted and customised for local needs.
- An outline of the potential costings of a new pain and frailty service or service components.

ETHICS AND DISSEMINATION

The POPPY study is a mixed-method study with low risk to participants. The POPPY study protocol was approved by the 28 April 2022 from The Yorkshire & the Humber - Leeds East Research Ethics Committee (Reference: 22/YH/0080). Consent is sought if an individual is willing to participate (Phases 2, 3 and 4) and has capacity.

The research will generate new knowledge and associated guidance for interventions, implementation strategies and services to improve access and deliver targeted support to reduce the pain burden of older adults living with frailty. Findings will be disseminated in numerous formats such as conferences, newsletters and journal papers and to a variety of groups including Local Authorities, Age UK, the British Pain Society and charities including Versus Arthritis.

DISCUSSION

For peer review only - http://bmjopen.bmj.com/site/about/guidelines.xhtml

The need to develop new models of care for older adults living with frailty is highlighted in the NHS Long Term Plan (12). In light of population ageing and the increasing numbers of people in advanced older age, targeted services addressing the specific needs of people with frailty are required. Older adults living with frailty are more likely to live with pain and for pain to have more impact on their lives than for fitter older adults (15). Pain can contribute to worsening frailty and an increase in incident frailty (17). As such, pain makes a suitable target for services for older adults. To date, we do not know the best way to deliver services for older adults living with both frailty and pain, where services should be situated to optimise uptake and adherence and what the components of a good pain and frailty service should include.

The POPPY study will use a mixed method co-design approach, working with relevant stakeholders to develop service and professional guidance to support older adults with frailty to better manage their pain A Patient and Public Involvement (PPI) group will contribute to all phases of the study. The POPPY study will include the views of an underserved group of older adults, i.e. those with frailty, often under-represented in research (19). Additionally, this study will include a range of different service models to understand the advantages and disadvantages of different service types to optimise take up and improve outcomes for this particular population.

Identifying POPPY participants from the CARE75+ cohort is an efficient and cost-effective way to identify a well-phenotyped population of older adults in terms of their frailty characteristics. The opportunity to capture additional information, from those that agree, from previously collected assessment data, including data on mobility, health conditions, loneliness and quality of life, will provide comprehensive contextual information to supplement interview findings. In-depth interviews will provide a detailed understanding of the impact of pain in this population, how they currently engage with services, and the reasons why some do not.

Some participants who participate in the interviews (phase 2) will be invited to co-design workshops (phase 4). Workshop participation will require a degree of engagement that may be beyond the scope of those living with dementia. As such those living with dementia have not been included in this study. However, we plan to share findings from Phase 2 and Phase 3 with at least one community dementia group to get their comments, insight and feedback. This will be shared with the stakeholder groups in the co-design workshops (Phase 4) so there is some recognition of the applicability of earlier findings to older adults living with dementia.

Older adults living in a care home or nursing home are not eligible for the POPPY study. We acknowledge that pain is an important issue, including for those living in care or nursing

For peer review only - http://bmjopen.bmj.com/site/about/guidelines.xhtml

homes (34). However, the typical characteristics of care home residents, often including a high prevalence of dementia, dependence for basic activities of daily living and very limited mobility, mean that service guidance would be different for this population. As such, this was beyond the scope of this study.

Furthermore, for Phase 2 of the POPPY study, older adults with a current cancer diagnosis are not eligible. Cancer survivors (at least 5 years cancer free and not undergoing active cancer treatment) can participate if they meet the eligibility criteria. Additionally, we will consult with the different services participating in Phase 3, on their guidelines for including cancer patients, or not, within their particular services.

The inclusion of a range of different pain services in different locations will enable us to consider what the best service models are to support older adults with frailty and pain. However, conducting research within NHS organisations presents challenges at the current time. This is partly as a legacy from COVID-19 and as a result of on-going capacity issues within NHS organisations. The POPPY study is a relatively low burden study for healthcare professionals to participate in, and the research team will work flexibly to accommodate service staff to minimise disruption to the service. Nevertheless, services are under pressure and some services may consider they lack the capacity to participate in research at this time due to service demands. As such, some pragmatic decisions may need to be made in terms of including those services that have the capacity to participate. We will make note of services that we approach that do not subsequently participate and the reasons why if these are provided.

** **

Contributors

LB: substantial contribution to the conception and design of the work; drafting the work and critical revisions; approval of final manuscript; accountable for all aspects of the work.
RM: substantial contribution to the conception and design of the work; drafting the work and critical revisions; approval of final manuscript; accountable for all aspects of the work.
NH: substantial contribution to the conception and design of the work; drafting the work and critical revisions; approval of final manuscript; accountable for all aspects of the work.
NL: substantial contribution to the conception and design of the work; drafting the work and critical revisions; approval of final manuscript; accountable for all aspects of the work.
AG: substantial contribution to the conception and design of the work; drafting the work and critical revisions; approval of final manuscript; accountable for all aspects of the work.
AC: substantial contribution to the conception and design of the work; drafting the work and critical revisions; approval of final manuscript; accountable for all aspects of the work.
AW: substantial contribution to the conception and design of the work; drafting the work and critical revisions; approval of final manuscript; accountable for all aspects of the work.

For peer review only - http://bmjopen.bmj.com/site/about/guidelines.xhtml

DA: substantial contribution to the conception and design of the work; drafting the work and critical revisions; approval of final manuscript; accountable for all aspects of the work.
PS: substantial contribution to the conception and design of the work; drafting the work and critical revisions; approval of final manuscript; accountable for all aspects of the work.
AS: substantial contribution to the conception and design of the work; drafting the work and critical revisions; approval of final manuscript; accountable for all aspects of the work.
AF: substantial contribution to the conception and design of the work; drafting the work and critical revisions; approval of final manuscript; accountable for all aspects of the work

Competing interests

1. All support for the present manuscript (e.g., funding, provision of study materials, medical writing, article processing charges, etc.): The POPPY study is funded by the National Institute for Health & Care Research (NIHR). Payment made to institutions for percentage of time/time contribution: LB, RM, NH, NL, AG, AC, AW, DA, PS, AS, AF.

2. Grants or contracts from any entity (if not indicated in item #1 above): DA is funded by a National Institute for Health and Care Research (NIHR) School for Primary Care Research post-doctoral award (project reference C075) and by a Chartered Society of Physiotherapy Charitable Trust post-doctoral award (Grant number PRF/21/POD01). Payments made to the Northern Care Alliance NHS Foundation Trust and University of Keele; PS has grant/contact with PEARN Charitable Trust. Payment made to University of Plymouth.

3. Royalties or licenses: none declared.

4. Consulting fees: none declared.

5. Payment or honoraria for lectures, presentations, speakers bureaus, manuscript writing or educational events. Royal College of General Practitioners Chair/Speaker fees to AS; Mibe Pharmaceuticals Speaker fees AS.

6. Payment for expert testimony: none declared.

7. Support for attending meetings and/or travel: none declared.

8. Patents planned, issued or pending: none declared.

9. Participation on a Data Safety Monitoring Board or Advisory Board: none declared.

10. Leadership or fiduciary role in other board, society, committee or advocacy group, paid or unpaid: MSK Champion Versus Arthritis Charity Leadership Role, AS.

11. Stock or stock options: none declared.

12. Receipt of equipment, materials, drugs, medical writing, gifts or other services: none declared.

13. Other financial or non-financial interests: none declared.

For peer review only

For peer review only - http://bmjopen.bmj.com/site/about/guidelines.xhtml

Funding

This project is funded by the National Institute for Health and Care Research (NIHR) Health Service and Delivery Research programme NIHR131319. The views expressed are those of the author(s) and not necessarily those of the NIHR or the Department of Health and Social Care.

References

1. Abdulla A, Adams N, Bone M, et al. Guidance on the management of pain in older adults. *Age Ageing* 2013; 42:i1-57.

2. Van Hecke O, Torrance N, Smith B. Chronic pain epidemiology and its clinical relevance. *Br. J. Anaesth* 2013; 111:13-8.

3. Fayaz A, Croft P, Langford RM, et al. Prevalence of chronic pain in the UK: a systematic review and meta-analysis of population studies. *BMJ Open* 2016; 6(6):e010364.

4. Chou KL. Reciprocal relationship between pain and depression in older adults: evidence from the English Longitudinal Study of Ageing. J. Affect. Disord 2007; 102:115-23.

5. Reid MC, Eccleston C, Pillemer K. Management of chronic pain in older adults. *BMJ* 2015;350.

6. Ong T, Thiam CN. Special consideration for pain management in the older person. *Clinical Medicine* 2022; 22:295.

7. Clegg A, Young J, Iliffe S, et al. Frailty in elderly people. Lancet 2013; 381:752–62.

8. O'Caoimh R, Sezgin D, O'Donovan MR, et al. Prevalence of frailty in 62 countries across the world: a systematic review and meta-analysis of population-level studies *Age Ageing* 2021; 50:96-104.

9. Rockwood K, Song X, MacKnight C, et al. A global clinical measure of fitness and frailty in elderly people. *CMAJ* 2005; 173:489-95.

10. Fried LP, Tangen CM, Walston J, et al. Frailty in older adults: evidence for a phenotype. *J Gerontol A Biol Sci Med Sci* 2001;56:M146–57.

11. Harrison JK, Clegg A, Conroy SP, et al. Managing frailty as a long-term condition. *Age Ageing* 2015; 44:732–5.

12. Department of Health. NHS Long term plan 2019 London: Department of Health; 2019. Available: https://www.longtermplan.nhs.uk. [Accessed 2023 Feb 07].

For peer review only - http://bmjopen.bmj.com/site/about/guidelines.xhtml

13. Clegg A, Bates C, Young J et al. Development and validation of an electronic frailty index using routine primary care electronic health record data. Age and ageing. 2016; 45:353-60.

14. Brown L, Young J, Clegg A, et al. Pain in older adults with frailty. *Reviews in Clinical Gerontology*. 2015; 25:159-71.

15. Brown L, Young J, Teale E. A cross-sectional study of the impact of pain in older people with frailty: findings from the community ageing research 75+ (CARE75+) study. Adv Geriatr Med Res

2019; 1:e190002.

16. Veronese N, Maggi S, Trevisan C, et al. Pain Increases the Risk of Developing Frailty in Older Adults with Osteoarthritis. *Pain Med*. 2017 ;18:414-427. doi: 10.1093/pm/pnw163. PMID: 27497322.

17. Saraiva MD, Suzuki GS, Lin SM, et al. Persistent pain is a risk factor for frailty: a systematic review and meta-analysis from prospective longitudinal studies. *Age Ageing* 2018; 47:785-93.

18. Crocker TF, Brown L, Clegg A, et al. Quality of life is substantially worse for community-dwelling older adults living with frailty: Systematic review and meta-analysis. *Quality of Life Research*. 2019:1-16.

19. National Institute for Health Research. Improving inclusion of under-served groups in clinical research: Guidance from INCLUDE project, 2020. Available: https://www.nihr.ac.uk/documents/improving-inclusion-of-under-served-groups-in-clinical-research-guidance-from-include-project/25435. [Accessed 2023 Feb 07].

20. Keefe FJ, Blumenthal J, Baucom D, et al. Effects of spouse-assisted coping skills training and exercise training in patients with osteoarthritic knee pain: a randomized controlled study. *Pain* 2004; 110:539-49.

21. Hackett J, Godfrey M, Bennett MI. Patient and caregiver perspectives on managing pain in advanced cancer: a qualitative longitudinal study. Palliative Medicine 2016; 30:711-9.

22. Calman L, Brunton L, Molassiotis A. Developing longitudinal qualitative designs: lessons learned and recommendations for health services research. BMC medical research methodology. 2013; 13:1-10.

23. Heaven A, Brown L, Young J, et al. Community ageing research 75+ study (CARE75+): an experimental ageing and frailty research cohort. *BMJ Open* 2019; 9:e026744.

For peer review only

For peer review only - http://bmjopen.bmj.com/site/about/guidelines.xhtml

24. Brown L, Heaven A, Quinn C, et al. Community Ageing Research 75+ (CARE75+) REMOTE study: a remote model of recruitment and assessment of the health, well-being and social circumstances of older adults. *BMJ Open*. 2021;11:e048524.

25. Treede R-D, Rief W, Barke A, et al. A classification of chronic pain for ICD-11. *Pain*. 2015; 156:1003.

26. Glaser BG, Strauss AL. The discovery of grounded theory: Strategies for qualitative research. Routledge; 2017.

27. National Institute for Health and Care Excellence (NICE). Chronic pain (primary and secondary) in over 16s: assessment of all chronic pain and management of chronic primary pain. London: 2021 [Accessed 2023 Feb 07]. Available from: https://www.nice.org.uk/guidance/ng193

28. Corbin, J Strauss, A. Basics of qualitative research. 3rd ed. London: Sage; 2008.

29. QSR International Pty Ltd. (2018) NVivo (Version 12), https://www.qsrinternational.com/nvivo-qualitative-data-analysis-software/home.

30. Harris PA, Taylor R, Thielke R, et al. Research electronic data capture (REDCap)—a metadata-driven methodology and workflow process for providing translational research informatics support. *Journal of biomedical informatics*. 2009; 42(2):377-81.

31. Harris PA, Taylor R, Minor BL, et al. The REDCap consortium: Building an international community of software platform partners. *Journal of biomedical informatics*. 2019; 95:103208.

32. Braun V, Clarke V. Using thematic analysis in psychology. *Qualitative research in psychology*. 2006; 3:77-101.

33. Ziebland S, Coulter A, Calabrese JD, Locock L. Editors. Understanding and using health experiences: improving patient experience. Oxford: Oxford University Press; c2013. Chapter 9, Participatory action research: using experience based co-design (EBCD) to improve healthcare services.

34. Schofield P. The assessment of pain in older people: UK national guidelines. *Age Ageing*. 2018 Mar 1; 47(suppl_1):i1-22.

For peer review only - http://bmjopen.bmj.com/site/about/guidelines.xhtml

Figure titles

Figure 1. Study flow diagram for the Pain in Older People with Frailty (POPPY) study

Figure 2. Study flow diagram for the identification and recruitment of participants for Phase 2 of the Pain in Older People with Frailty (POPPY) study

For peer review only

For peer review only - http://bmjopen.bmj.com/site/about/guidelines.xhtml

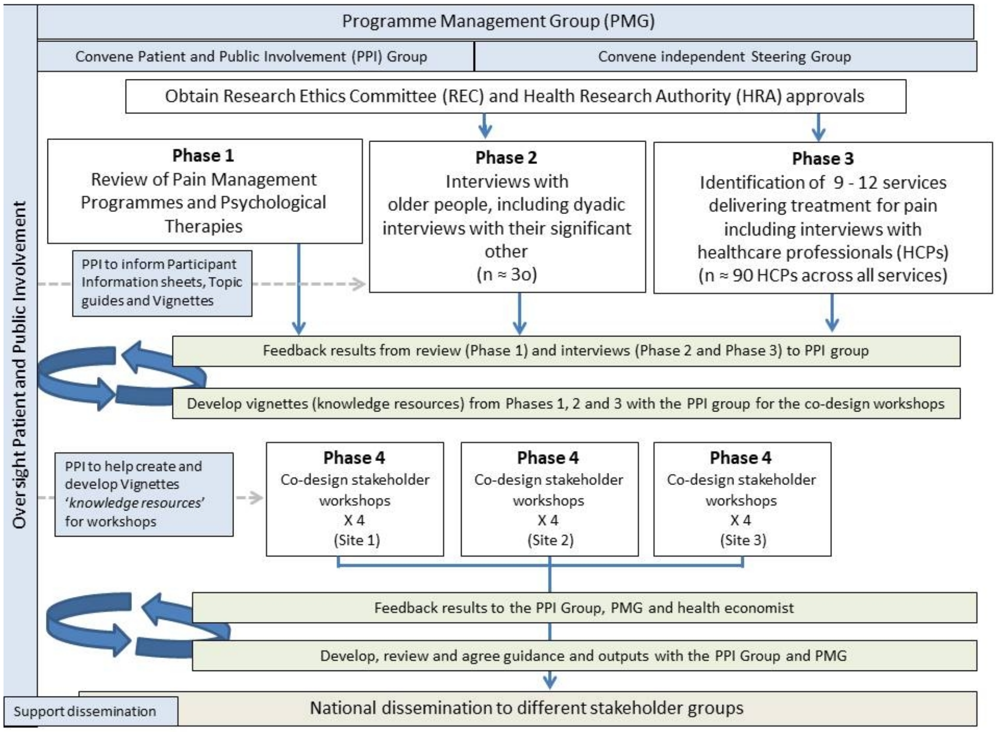

Study flow diagram for the Pain in Older People with Frailty (POPPY) study

254x190mm (96 x 96 DPI)

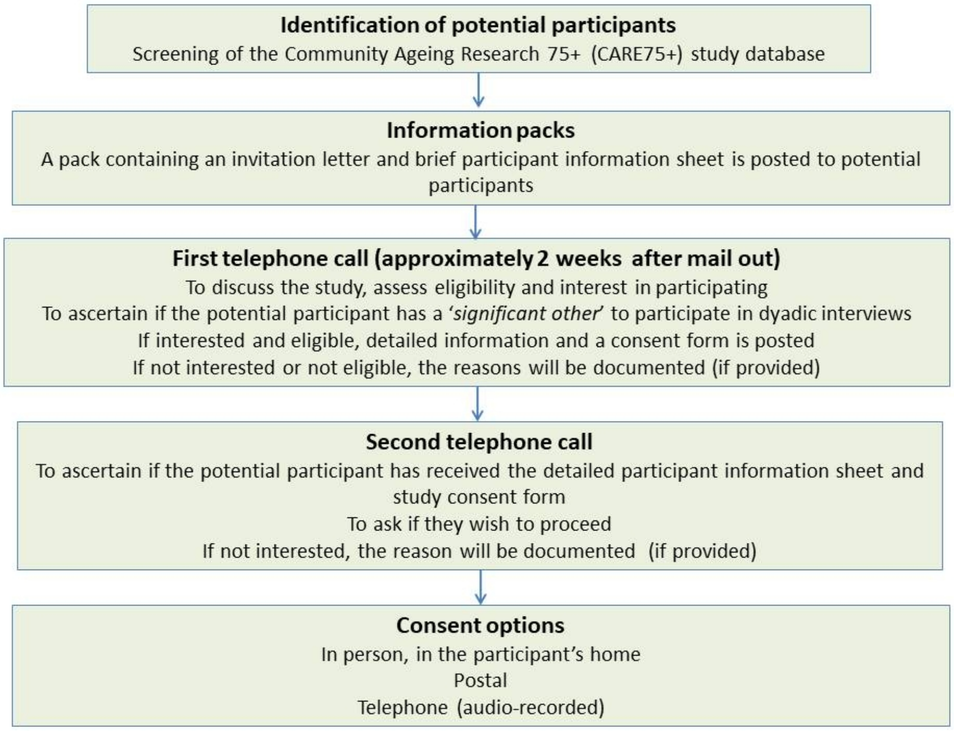

Study flow diagram for the identification and recruitment of participants for Phase 2 of the Pain in Older People with Frailty (POPPY) study

254x190mm (96 x 96 DPI)
