# Supplementary material for: Developing the evidence and associated service models to support older adults living with frailty to manage their pain and to reduce its impact on their lives: protocol for a mixed-method, co-design study (The POPPY Study)
Source: BMJ Open. 2023 Jun 26;13(6):e074785. doi: 10.1136/bmjopen-2023-074785 (PMC10314646; doi:10.1136/bmjopen-2023-074785)
Supplement: Reviewer comments [file bmjopen-2023-074785.reviewer_comments.pdf]

PEER REVIEW HISTORY

BMJ Open publishes all reviews undertaken for accepted manuscripts. Reviewers are asked to complete a checklist review form (**http://bmjopen.bmj.com/site/about/resources/checklist.pdf**) and are provided with free text boxes to elaborate on their assessment. These free text comments are reproduced below.

ARTICLE DETAILS

**TITLE (PROVISIONAL)**	Developing the evidence and associated service models to support older adults living with frailty to manage their pain and to reduce its impact on their lives: protocol for a mixed-method, co-design study (The POPPY Study)
**AUTHORS**	Brown, Lesley; Mossabir, Rahena; Harrison, Nicola; Lam, Natalie; Grice, Anne; Clegg, Andrew; Williams, Amanda; Antcliff, Deborah; Schofield, Patricia; Suleman, Asim.; Forster, Anne

This article was not externally reviewed at BMJ Open. Protocols that have been independently assessed prior to submission to BMJ Open are usually fast-tracked to publication on the grounds that further substantial changes will not be possible. This independent assessment will usually be external, independent review for both a substantial grant award from a non-commercial or government funder and ethics approval.
